# Alanyl-Glutamine Dipeptide Attenuates Non-Alcoholic Fatty Liver Disease Induced by a High-Fat Diet in Mice by Improving Gut Microbiota Dysbiosis

**DOI:** 10.3390/nu15183988

**Published:** 2023-09-14

**Authors:** Yigang Zheng, Hanglu Ying, Jiayi Shi, Long Li, Yufen Zhao

**Affiliations:** 1 Institute of Drug Discovery Technology, Ningbo University, Ningbo 315211, China; 2111074064@nbu.edu.cn (Y.Z.); 1911074039@nbu.edu.cn (H.Y.); 2111074037@nbu.edu.cn (J.S.); zhaoyufen@nbu.edu.cn (Y.Z.); 2 Qian Xuesen Collaborative Research Center of Astrochemistry and Space Life Sciences, Ningbo University, Ningbo 315211, China

**Keywords:** alanyl-glutamine, non-alcoholic fatty liver disease, gut microbiota, lipid accumulation, inflammation

## Abstract

Non-alcoholic fatty liver disease (NAFLD) manifests as a persistent liver ailment marked by the excessive buildup of lipids within the hepatic organ accompanied by inflammatory responses and oxidative stress. Alanyl-glutamine (AG), a dipeptide comprising alanine and glutamine, is commonly employed as a nutritional supplement in clinical settings. This research aims to evaluate the impact of AG on NAFLD triggered by a high-fat diet (HFD), while concurrently delving into the potential mechanisms underlying its effects. The results presented herein demonstrate a notable reduction in the elevated body weight, liver mass, and liver index induced by a HFD upon AG administration. These alterations coincide with the amelioration of liver injury and the attenuation of hepatic histological advancement. Furthermore, AG treatment manifests a discernible diminution in oil-red-O-stained regions and triglyceride (TG) levels within the liver. Noteworthy alterations encompass lowered plasma total cholesterol (TC) and low-density lipoprotein cholesterol (LDLC) concentrations, coupled with elevated high-density lipoprotein cholesterol (HDLC) concentrations. The mitigation of hepatic lipid accumulation resultant from AG administration is aligned with the downregulation of ACC1, SCD1, PPAR-γ, and CD36 expression, in conjunction with the upregulation of FXR and SHP expression. Concomitantly, AG administration leads to a reduction in the accumulation of F4/80-positive macrophages within the liver, likely attributable to the downregulated expression of MCP-1. Furthermore, AG treatment yields a decline in hepatic MDA levels and a concurrent increase in the activities of SOD and GPX. A pivotal observation underscores the effect of AG in rectifying the imbalance of gut microbiota in HFD-fed mice. Consequently, this study sheds light on the protective attributes of AG against HFD-induced NAFLD through the modulation of gut microbiota composition.

## 1. Introduction

Non-alcoholic fatty liver disease (NAFLD) is a medical condition defined by the accumulation of lipid deposits in the liver without the involvement of alcohol abuse, which is a prevalent chronic liver ailment that has emerged as a significant global public health challenge [1]. Current estimations indicate that 25% of the worldwide population is afflicted with NAFLD, and the incidence of this condition is experiencing a worrisome upward trajectory [2]. NAFLD encompasses a spectrum of liver disorders, spanning from simple hepatic steatosis to more severe non-alcoholic steatohepatitis (NASH), which has the potential to progress to liver cirrhosis and hepatocellular carcinoma (HCC) [3]. Pathologically, NAFLD is characterized by the presence of fat within hepatocytes, inflammation, and fibrosis. The development and progression of NAFLD are intricate and involve a combination of genetic, metabolic, and environmental factors. Central to its pathogenesis are mechanisms such as oxidative stress, insulin resistance, and lipid accumulation [4]. Presently, there is no specific remedy exclusively designed for NAFLD. The main therapeutic strategies include dietary adjustments and physical exercise, which aim to ameliorating insulin resistance and diminish liver lipid accumulation [5]. Nevertheless, significant challenges remain in the diagnosis and management of NAFLD. These challenges include the absence of accurate and dependable diagnostic tools, the absence of effective pharmacological treatments, and the need for a more comprehensive understanding of the molecular underpinnings governing the pathogenesis of NAFLD [6]. Thus, more research efforts are urgently needed to address these issues and develop effective preventive and therapeutic interventions for NAFLD.

Recent research has provided compelling evidence linking gut dysbiosis to NAFLD, denoting an irregularity in the composition of the gut microbiota [7]. Furthermore, the gut microbiota is involved in the regulation of gut permeability, which is notably increased in patients afflicted with NAFLD. Heightened gut permeability leads to the migration of bacterial endotoxins including lipopolysaccharides (LPS) into the systemic circulation, where they can trigger inflammation and contribute significantly to the pathogenesis and progression of NAFLD [8]. Additionally, recent researches have demonstrated that interventions targeting the gut microbiota, such as prebiotics, probiotics, and fecal microbiota transplantation (FMT), can improve NAFLD-related outcomes by modulating gut dysbiosis and reducing gut permeability and inflammation [9]. Given the pivotal influence of the gut microbiota on the onset and advancement of NAFLD, directing therapeutic efforts toward modulating the gut microbiota using pharmacological agents emerges as a promising avenue for both preventing and managing this condition [10].

Alanyl-glutamine dipeptide (AG) is a dipeptide composed of alanine and glutamine that has gained attention in recent years for its biological functions and potential therapeutic applications in various diseases [11]. AG could act as a source of glutamine, a crucial nutrient for cellular metabolism and immune function. Previous research has extensively demonstrated the pivotal significance of AG in maintaining the integrity and function of the mucosal barrier within the gastrointestinal tract, while enhancing the immune response, making it a highly appealing contender for the prevention and therapeutic management of gastrointestinal disorders, such as inflammatory bowel disease (IBD) and acute pancreatitis [12]. Additionally, AG has exhibited potential neuroprotective benefits across diverse experimental frameworks involving neurological disorders, encompassing traumatic brain injuries, strokes, and conditions marked by neurodegeneration [13]. Moreover, AG has been found to have beneficial effects on liver function, including hepatoprotection and the attenuation of liver injury induced by oxidative stress, making it a promising therapeutic agent for various liver diseases [14,15]. We have reported the therapeutic potential of AG in methionine- and choline-deficient (MCD) diet-triggered NASH [16]. However, the MCD diet-treated mice are lean mice which could reflect the pathological features of obesity-related NAFLD; whether AG could affect HFD-induced obesity-related NAFLD still needs to be studied further.

This current investigation delved into the involvement of AG in the advancement of NAFLD caused by HFD, shedding light on its interaction with gut microbiota during this process. The data reveal AG’s protective influence in mitigating HFD-induced NAFLD in the murine model through modulating the gut microbiota composition.

## 2. Materials and Methods

### 2.1. Materials

AG was obtained as previously reported [16]. The standard diet (SD) and HFD (60 kcal%) was obtained from Research Diets Inc. (New Brunswick, NJ, USA). The compositions of the SD and HFD are shown in Supplementary Table S1.

### 2.2. Animal Experiments

Male C57BL/6 mice, within the age range of 6–8 weeks, were procured from Vital River Laboratory Animal Technology Co., Ltd. (Beijing, China), with a weight ranging from 21 g to 25 g. The mice were accommodated within a specific pathogen-free (SPF) environment characterized by consistent temperature (21–23 °C) and humidity (50–60%). These conditions were maintained under a 12 h light/dark cycle, with ad libitum access to both food and water of high purity. Following a week of acclimatization, the mice were subject to random allocation into two initial groups: a normal diet group (ND) and a high-fat diet group (HFD). After a span of 20 weeks, the mice were once again randomly redistributed into three groups (*n* = 6–8/group), as delineated below: (a) a control group receiving a normal diet, (b) a model group exposed to an HFD along with a daily gavage of saline, and (c) an AG treatment group exposed to an HFD supplemented with a daily gavage of 1500 mg/kg AG. The mice were scheduled for euthanasia after four weeks. Subsequent analysis necessitated the collection of blood, liver, epididymal white adipose tissue (WAT), and fecal samples. After the liver mass, WAT mass, and body weight were weighed, the liver index and WAT index were calculated as liver mass/body weight and WAT mass/body weight. All animal experimental protocols received ethical approval from the Animal Ethics Committee of Ningbo University (Approved No. NBU20220123) and were executed in adherence to the established guidelines governing the utilization of animals in research.

### 2.3. Histology

Liver specimens were subjected to fixation using 4% paraformaldehyde (Solarbio, Beijing, China) for a duration of 24 h. Subsequent to fixation, the specimens underwent paraffin embedding, following which they were sectioned into 5 μm slices of hepatic tissue. These slices subsequently underwent hematoxylin-eosin (HE) (Solarbio, Beijing, China) staining to facilitate the assessment of pathological modifications. Additionally, oil red O (ORO) (Merck, Shanghai, China) staining was employed on frozen liver sections to facilitate the visualization of lipid accumulation within the hepatic tissue.

### 2.4. IHC Assay

Liver paraffin sections underwent deparaffinization and hydration processes, after which they were subjected to antigen retrieval through citrate buffer (Solarbio, Beijing, China) boiling. Subsequent steps included the treatment of sections with 0.3% hydrogen peroxide (Sinopharm Chemical Reagent Co., Ltd., Shanghai, China), followed by blocking utilizing 3% goat serum (Solarbio, Beijing, China). The incubation process involved an overnight treatment with an antibody against F4/80 (Abcam, Shanghai, China), which was conducted at a temperature of 4 °C. Following this, several PBS washes were performed before proceeding to a secondary antibody incubation. After a subsequent wash, the sections were subjected to staining using 3,3-diaminobenzidine (DAB) (Solarbio, Beijing, China), and then counterstaining with hematoxylin (Solarbio, Beijing, China). The acquisition of images was facilitated using a Leica optical microscope (Shanghai, China).

### 2.5. Plasma and Liver Chemistry

After blood collection, the samples were transferred to centrifuge tubes containing EDTA. Centrifugation was then performed to separate the plasma from the cellular components at 3000× *g* for 15 min at 4 °C. The plasma samples were obtained as the clear, yellowish fluid that remains on top after centrifugation. The concentrations of ALT, AST, LDH, LDLC, HDLC, TG, and TC were quantified within mouse plasma. These determinations were executed employing kits sourced from the Nanjing Jiancheng Bioengineering Institute (Nanjing, China). Concomitantly, the assessment of MDA, SOD, and GPX levels within the mouse liver was carried out through the utilization of commercial biochemical kits, also sourced from the Nanjing Jiancheng Bioengineering Institute. The quantification of hepatic TG content was achieved using a TG assay kit (BioVision Inc., Milpitas, CA, USA).

### 2.6. Real-Time PCR

RNA extraction and subsequent reverse transcription procedures were carried out using kits procured from TianGen Biotech Co., Ltd. (Beijing, China). Following the acquisition of cDNA, the quantification of mRNA was executed employing the SYBR Green kit, also from TianGen Biotech Co., Ltd. (Beijing, China). The target genes measured using real-time PCR included ACC1, SCD1, PPAR-γ, CD36, FXR, SHP, and MCP-1. To facilitate appropriate normalization, the obtained experimental outcomes were standardized against the expression of the housekeeping gene GAPDH.

### 2.7. Gut Microbiota Analyses

Fresh fecal specimens were meticulously collected and immediately stored within sterile tubes, maintaining a storage temperature of −80 °C for subsequent analysis. The extraction of total microbial DNA from these fecal samples was carried out using a commercial DNA extraction kit obtained from Omega Bio-Tek Inc. (Norcross, GA, USA). The resultant DNA’s concentration and purity were subsequently gauged utilizing a NanoDrop device (NanoDrop Technologies, Wilmington, DE, USA). PCR was employed to amplify the V3–V4 region of the 16S rRNA gene in this study utilizing universal bacterial primers. The resulting purified PCR products were then utilized for library construction, which was subsequently subjected to sequencing on the Illumina NovaSeq platform. Rigorous quality control measures, sequence filtration, the clustering of Operational Taxonomic Units (OTUs), species annotation, and diversity analyses were systematically applied to the sequenced data. These comprehensive analyses served to investigate the diversity exhibited by the gut microbiota. 

### 2.8. Elisa

The MCP-1 level in plasma was measured using an ELISA kit from MultiSciences Biotech (Hangzhou, China).

### 2.9. Data Analysis

The data collected were analyzed using GraphPad Prism software (version 8.3.0). The results were presented as the mean ± SEM. To assess differences among multiple groups, a one-way analysis of variance (ANOVA) was performed. *p* < 0.05 was deemed indicative of statistical significance.

## 3. Results

### 3.1. Effect of AG Administration on Body Weight, Liver Mass, and Liver Index in HFD-Induced Mice

**Figure 1:**
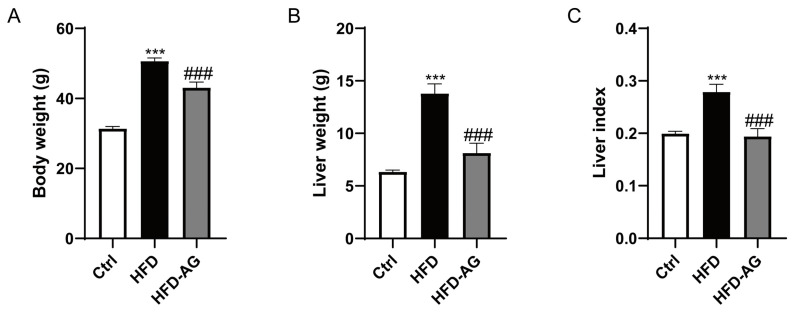
Effect of AG on the body weight, liver mass, and liver index in HFD-feeding mice. (**A**) Body weight. (**B**) Liver mass. (**C**) Liver index. Results are presented as the mean ± SEM. *** *p* < 0.001 vs. Ctrl, ### *p* < 0.001 vs. HFD.

The interconnection between obesity and the advancement of diverse metabolic disorders, including NAFLD, is well established [17]. In this study, obesity in murine subjects was induced through a 20-week high-fat diet (HFD) regimen. The HFD regimen notably precipitated marked increases in body weight, liver mass, liver index, and mass of WAT. Remarkably, treatment with AG displayed a significant mitigation in the HFD-induced elevation of body weight, liver mass, and liver index (Figure 1). Additionally, the mass of epididymal WAT was measured, revealing a substantial increase due to HFD feeding. However, it is noteworthy that AG treatment did not exert a significant impact on WAT weight or WAT index in the context of HFD-induced mice (Supplementary Figure S1).

### 3.2. Effects of AG Treatment on HFD-Induced Liver Damage in Mice

**Figure 2:**
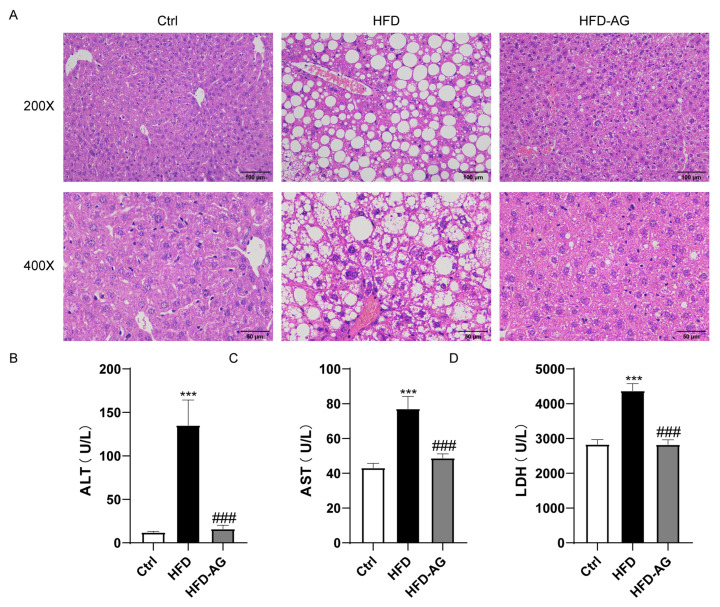
Effect of AG on histopathology and plasma biomarkers in HFD-treated mice. (**A**) Representative liver histological images of HE staining (original magnification of 200× and 400×); (**B**) ALT; (**C**) AST; (**D**) LDH. Results are presented as the mean ± SEM. *** *p* < 0.001 vs. Ctrl, ### *p* < 0.001, vs. HFD.

Figure 1A illustrates the outcome of HFD feeding, which incited hepatic steatosis along with mild inflammatory responses within the liver. However, AG treatment effectively mitigated these pathological alterations (Figure 2A). Notably, plasma concentrations of ALT, AST, and LDH exhibited significant elevation in mice subjected to HFD feeding, contrasting with those on a normal diet. Impressively, AG supplementation yielded a marked reduction in ALT, AST, and LDH levels, underscoring the evident protective impact of AG against HFD-induced hepatic injury (Figure 2B–D). 

### 3.3. Attenuation of Hepatic Lipid Accumulation via AG Supplementation in HFD-Induced Mice

**Figure 3:**
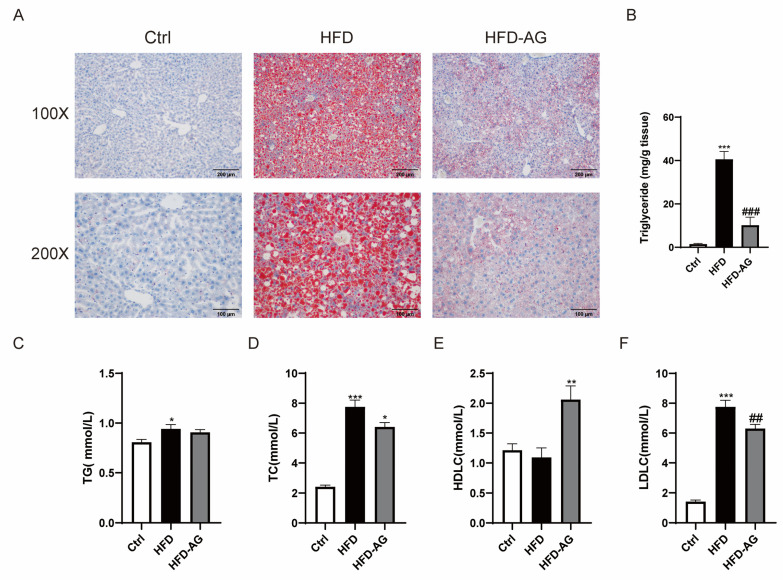
Effect of AG on liver steatosis in NAFLD mice. (**A**) Representative images of ORO staining in liver sections (original magnification of 100× and 200×). (**B**) Liver TG levels. (**C**) Plasma TG levels. (**D**) Plasma TC levels. (**E**) Plasma HDLC levels. (**F**) Plasma LDLC levels. Results are presented as the mean ± SEM. * *p <* 0.05, ** *p <* 0.01, *** *p <* 0.001 vs. Ctrl, ## *p <* 0.01, ### *p <* 0.001 vs. HFD.

Observations stemming from the ORO staining of liver sections distinctly portray a notable buildup of lipid droplets within the livers of HFD-treated mice. Remarkably, AG treatment engendered a substantial alleviation of hepatic lipid accumulation (Figure 3A). Correspondingly, hepatic TG levels exhibited a considerable increase within the HFD group as opposed to the control group. It is noteworthy that AG treatment conferred a reduction in hepatic TG levels (Figure 3B).

The investigation extended to include the assessment of TG, TC, LDLC, and HDLC levels within plasma. Although plasma TG levels displayed a conspicuous increase within the HFD group, no appreciable distinction was discerned between the HFD cohort and the HFD-AG group (Figure 3C). It is worth highlighting the intriguing outcome: AG treatment significantly counteracted the HFD-induced elevation in both TC and LDLC levels (Figure 3D,E). Notably, AG administration also yielded a significant upregulation of HDLC levels within the plasma when compared with the HFD group (Figure 3F).

### 3.4. Impact of AG on Fatty-Acid-Metabolism-Related Genes in NAFLD Mice

**Figure 4:**
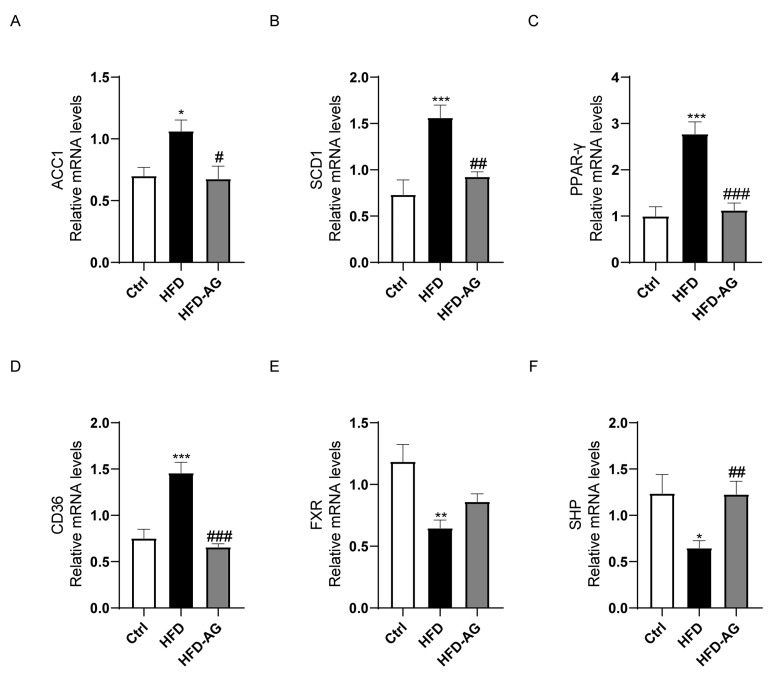
Effect of AG on hepatic expression of fatty-acid-metabolism-related genes in HFD-induced NAFLD mice. (**A**) ACC1; (**B**) SCD1; (**C**) PPAR-γ; (**D**) CD36; (**E**) FXR; (**F**) SHP. Results are presented as the mean ± SEM. * *p <* 0.05, ** *p <* 0.01, *** *p <* 0.001 vs. Ctrl, # *p <* 0.05, ## *p <* 0.01, ### *p <* 0.001 vs. HFD group.

To unveil the underlying molecular pathways responsible for the lipid-lowering attributes of AG within the hepatic tissue of HFD-treated NAFLD mice, we investigated the mRNA expression patterns pertaining to a repertoire of pivotal genes associated with fatty acid metabolism, namely ACC1, SCD1, PPAR-γ, CD36, FXR, and SHP. The findings indicated a substantial elevation in mRNA levels of ACC1, SCD1, PPAR-γ, and CD36 within the HFD group as opposed to the control group. Notably, AG intervention exhibited a pronounced ameliorative effect, effectively mitigating these upregulations (Figure 4A–D).

Furthermore, HFD feeding elicited a noteworthy reduction in the mRNA expression of FXR and SHP. While no significant alterations were observed in FXR expression between the HFD group and the HFD-AG group, AG treatment yielded a marked enhancement in SHP expression (Figure 4E,F).

### 3.5. Mitigation of Macrophage Accumulation by AG in HFD-Treated Mice

**Figure 5:**
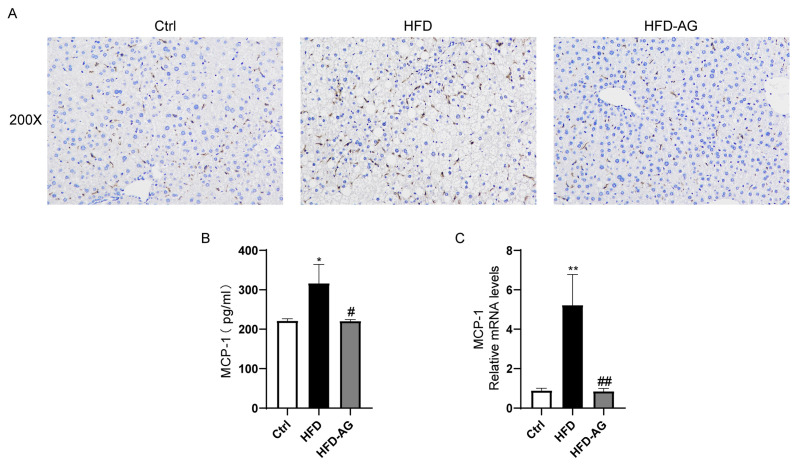
AG suppressed hepatic macrophage accumulation in HFD-induced NAFLD mice. (**A**) IHC analysis of F4/80. (**B**) MCP-1 protein concentrations in plasma. (**C**) Hepatic MCP-1 mRNA expression. Results are presented as the mean ± SEM. * *p <* 0.05, ** *p <* 0.01 vs. Ctrl, # *p <* 0.05, ## *p <* 0.01, vs. HFD.

To explore the profile of inflammatory cell infiltration within the liver upon HFD feeding and AG intervention, an F4/80 IHC analysis was executed. The outcomes revealed a substantial escalation in the presence of F4/80-positive macrophages within the livers of mice belonging to the HFD experimental group in comparison to the control group. Notably, AG treatment exerted a robust reduction in the abundance of macrophages accumulated in the liver. This underscores AG’s potential in curbing macrophage infiltration within the liver (Figure 5A).

A key chemokine, MCP-1, known for its role in promoting macrophage accumulation, has garnered attention [18]. In this study, HFD feeding clearly engendered an upregulation in MCP-1 mRNA levels within the liver, and correspondingly elevated the protein levels of MCP-1 within the plasma, discernibly in contrast to the control group. Importantly, AG treatment has emerged as a pivotal mediator in significantly curbing MCP-1 levels, both within the liver and plasma (Figure 5B).

### 3.6. Hepatic Oxidative Stress Mitigation by AG in HFD-Induced Mice

**Figure 6:**
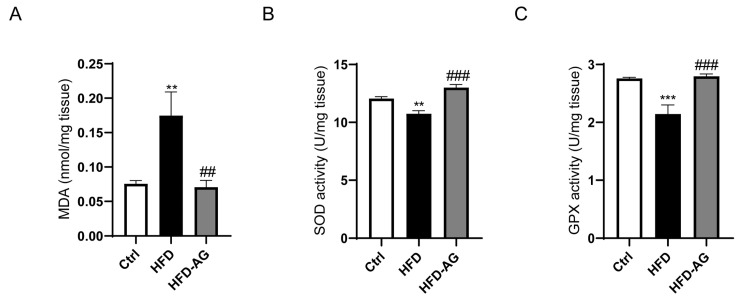
Effect of AG on hepatic oxidative stress in HFD-fed mice. (**A**) The content of MDA. (**B**) The activities of SOD. (**C**) The activities of GPX. Results are presented as the mean ± SEM. ** *p <* 0.01, *** *p <* 0.001 vs. Ctrl, ## *p <* 0.01, ### *p <* 0.001 vs. HFD.

Oxidative stress, recognized as a crucial exacerbating factor exacerbating the progression of NAFLD, warrants thorough exploration [19]. In light of this, the potential influence of AG on hepatic oxidative stress was investigated. The assessment encompassed pertinent oxidative stress parameters, unveiling a noteworthy upregulation in MDA levels and a discernible reduction in the activities of SOD and GPX within the HFD group, when contrasted with the control group. Significantly, AG administration precipitated a remarkable reversal of these perturbations, effectively reinstating the equilibrium (Figure 6). 

### 3.7. Effect of AG on Gut Microbiota Profiles in HFD-Fed Mice

**Figure 7:**
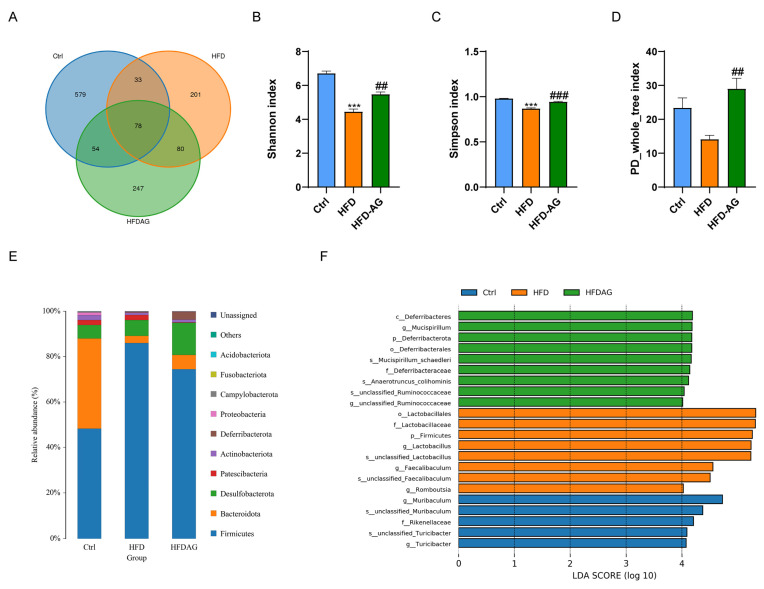
AG modulates gut microbiota against dysbiosis triggered by HFD. (**A**) Venn diagram. (**B**) Shannon index. (**C**) Simpson index. (**D**) PD_whole_tree index. (**E**) Composition of gut microbiota at the phylum level. (**F**) LEfSe analysis shows the key bacteria response to HFD and AG treatment at the genus level. *** *p <* 0.001 vs. Ctrl, ## *p <* 0.01, ### *p <* 0.001 vs. HFD.

The pivotal role of gut microbiota in the trajectory of NAFLD development has been widely acknowledged [20]. To elucidate the regulatory role of AG in the growth of the intestinal microbiota, we initially employed LC-MS to ascertain whether AG could reach the colon and rectal regions following administration. The results revealed the presence of AG in the fecal samples collected 2 and 6 h after the oral gavage of AG (1500 mg/kg), indicating its successful delivery to these regions (Supplementary Figure S2). In this context, the influence of AG on gut microbiota within HFD-fed mice was meticulously explored through 16S rRNA sequencing. The ensuing Venn diagram (Figure 7A) deftly illustrates the common and distinctive operational taxonomic units (OTUs) prevalent across differential groups. Of notable significance are the indexes of Shannon, Simpson, and PD_whole_tree, which exhibited a palpable reduction subsequent to HFD feeding in mice. In stark contrast, AG administration decisively elevated these indexes. This trend implies AG’s potential to mitigate the diminished abundance and diversity of gut microbiota induced by HFD feeding (Figure 7B–D).

The evaluation extended further to encompass the relative abundances of bacteria. The analysis divulged the dominance of *Firmicutes*, *Bacteroidota*, *Desulfobacterota*, and *Patescibacteria* at the phylum level. Remarkably, the relative abundance of *Firmicutes* registered a noticeable upregulation upon HFD feeding. Encouragingly, AG intervention substantially counteracted this upregulation (Figure 7E). A rigorous evaluation of bacterial abundance across diverse groups was conducted through LEfSe analysis. This revealed substantial alterations in the relative abundances of 5, 8, and 9 bacterial species within the control, HFD, and HFD-AG groups, respectively (Figure 7F). Together, the accumulated data allude to AG’s potential in modulating the composition of gut microbiota within the context of HFD-fed mice.

## 4. Discussion

During an era of rapid economic growth, NAFLD has quickly emerged as one of the prevailing chronic liver ailments worldwide. The HFD-induced NAFLD mouse model is currently one of the most frequently used models [21]. Prolonged consumption of an HFD can cause lipid deposition, hepatic oxidative stress, and inflammatory response in mice, while also leading to an imbalance in the gut microbiota, mirroring the development process of NAFLD in humans [22]. In the scope of our investigation, we observed that the administration of AG yielded substantial reductions in body weight, liver mass, and the liver-to-body weight ratio in mice subjected to an HFD. Additionally, it improved hepatic cell damage, lipid deposition within the liver, oxidative stress, and inflammation induced by the HFD. Furthermore, our results revealed that AG restored the dysbiosis within the gut microbiota in NAFLD mice. These findings suggest that AG may serve as a potential therapeutic modality in the management of NAFLD. 

The escalation of energy intake can precipitate a derangement in energy metabolism, ultimately fostering lipid accumulation within both adipose tissue and the liver, culminating in obesity [23]. Mice subjected to HFD, mirroring obesity and its concomitant disorders such as NAFLD, have become a frequently adopted animal model [24]. In consonance with precedent research, our investigations showcased a substantial rise in body weight, adipose tissue mass, and liver weight upon extended HFD consumption. Notably, AG treatment effectuated a marked reduction in both body weight and liver weight. Remarkably, this influence did not extend to adipose tissue mass, indicating a nuanced mechanism at play in AG’s capacity to counteract the HFD-induced elevation in liver weight, potentially dissociated from the adipose tissue.

The pivotal involvement of free fatty acids in hepatic TG accumulation is well recognized, stemming from de novo lipogenesis or external uptake [3]. Among the key enzymes governing fatty acid biosynthesis, ACC1 and SCD-1 are paramount [25]. Additionally, the transcription factor PPAR-γ exerts substantial influence over lipid metabolism and fatty acid uptake, and its prominence in hepatic steatosis is well-documented [26]. The transmembrane glycoprotein, CD36, facilitates the assimilation and utilization of exogenous fatty acids via hepatocytes [27]. Evidently, HFD exposure elicited a notable elevation in the expression of ACC1, SCD-1, PPAR-γ, and CD36 genes within the murine liver. In a significant reversal, AG administration substantially curtailed the expression of these genes. Another pivotal player, the nuclear receptor FXR, assumed a key role in fatty acid metabolism [28]. Concomitantly, the downstream mediator of the FXR signaling pathway, SHP, exhibited discernible involvement [29]. A cascade emerged wherein SHP’s upregulation correlated with the inhibition of ACC1 transcription, thus abating fatty acid synthesis [30]. Notably, AG treatment conspicuously augmented the expression of SHP within the murine liver, reinforcing the potential of AG in the attenuation of fatty acid synthesis and uptake.

One of the primary characteristics of NAFLD involves the abnormal buildup of fat within the hepatic tissue, resulting in the excessive generation of mitochondrial reactive oxygen species (ROS) [31]. Antioxidant status is typically compromised in NAFLD patients, which is manifested through elevated levels of lipid peroxidation byproducts and systemic markers indicative of oxidative stress [32]. Therefore, the regulation of antioxidant response becomes a promising avenue for preventing the occurrence and progression of NAFLD [33]. In alignment with previous findings, our study discerned a noteworthy escalation in MDA content and a great decrease in the contents of SOD and GPX in the hepatic tissue of NAFLD mice. However, treatment with AG significantly increased the activity of these two antioxidant enzymes and markedly reduced the MDA content. These results suggest that the protective influence of AG on NAFLD might be partially ascribed to its antioxidative function.

Increasing evidence suggests that liver cell injury and steatosis in the progression of NAFLD are associated with inflammation, which exacerbates the transition from NAFLD to NASH [34]. Previous studies have indicated that the depletion of Kupffer cells in mice subjected to an HFD yielded a notable reduction in hepatic steatosis and inflammation [35]. In our results, we observed a significant reversal of macrophage accumulation in the liver induced by an HFD following AG treatment. Additionally, AG exhibited inhibitory effects on the expression of MCP-1 in both mouse plasma and liver. MCP-1 plays a crucial role in recruiting macrophages during the inflammatory process [36]. Therefore, AG may improve the inflammatory status of the liver by suppressing MCP-1 expression.

Gut microbiota dysbiosis emerges as a pivotal risk factor in the HFD-driven progression of NAFLD [37]. Reports indicate that HFD-driven NAFLD in mice is accompanied by a decrease in bacterial diversity [38]. Consistent with previous studies, our results showed that the Shannon, Simpson, and PD_whole_tree indexes of the gut microbiota were reduced in mice fed with HFD compared to the control group. Noteworthy, AG treatment surged these indices, underscoring its potential in counteracting HFD-induced gut microbiota diversity reduction. Aligning with precedent research, an augmented *Firmicutes* abundance was apparent in HFD-induced obese mice, paralleled in our study wherein *Firmicutes* dominance was markedly elevated in the HFD group in contrast to the control group [39]. Intriguingly, AG intervention yielded a partial reversal of this heightened *Firmicutes* abundance. Collectively, these insights accentuate AG’s potential in rectifying obesity-related gut dysbiosis consequential to HFD, further engendering a favorable impact on NAFLD pathogenesis.

In our current investigation, AG appears to ameliorate liver steatosis through a multifaceted mechanism. It likely reduces the accumulation of lipids in the liver by regulating key components of lipid metabolism. Currently, AG does not have recognized target proteins; our research and previous studies have not identified any specific protein with which AG can directly interact. Notably, the nuclear receptor FXR stands out as a promising target for NAFLD therapy [28]. Our findings reveal that AG has the capacity to upregulate FXR expression, concomitantly elevating the expression of the downstream gene SHP. Consequently, we speculate that AG holds the potential to directly modulate FXR, thereby regulating lipid metabolism. Nevertheless, further experimentation is imperative to corroborate this hypothesis. Furthermore, our data demonstrated that AG exhibits a multifaceted approach to enhance liver steatosis, encompassing gene expression regulation, anti-inflammatory and antioxidant properties, and the modulation of the gut microbiota. While the precise molecular targets and macromolecular interactomes involved necessitate additional investigation, these mechanisms collectively underlie the beneficial effects of AG in attenuating hepatic steatosis induced by an HFD.

## 5. Conclusions

In summary, the administration of AG led to reductions in body weight, liver mass, and the liver-to-body weight ratio. Furthermore, it resulted in improvements in liver injury, steatosis, inflammation, and oxidative stress among mice afflicted with NAFLD due to HFD consumption. These beneficial effects are likely attributed to AG’s capacity to modulate the gut microbiota. Consequently, these findings offer fresh perspectives on the promising utility of AG administration as a prospective approach to prevent and treat NAFLD.

## Data Availability

The data presented in this study are available on request from the corresponding author.

## Supplementary Materials

The following supporting information can be downloaded at: https://www.mdpi.com/article/10.3390/nu15183988/s1, Figure S1: Effects of AG on the white adipose tissue weight and white adipose tissue index in HFD feeding mice; Figure S2: LC-MS analysis of AG in fecal samples. Table S1: Composition of the standard diet (SD) and HFD diet formulas.

## References

[1] Abdelmalek M.F. (2021). Nonalcoholic fatty liver disease: Another leap forward. Nat. Rev. Gastroenterol. Hepatol..

[2] Fan J.G., Kim S.U., Wong V.W. (2017). New trends on obesity and NAFLD in Asia. J. Hepatol..

[3] Ipsen D.H., Lykkesfeldt J., Tveden-Nyborg P. (2018). Molecular mechanisms of hepatic lipid accumulation in non-alcoholic fatty liver disease. Cell Mol. Life Sci..

[4] Younossi Z.M., Loomba R., Rinella M.E., Bugianesi E., Marchesini G., Neuschwander-Tetri B.A., Serfaty L., Negro F., Caldwell S.H., Ratziu V. (2018). Current and future therapeutic regimens for nonalcoholic fatty liver disease and nonalcoholic steatohepatitis. Hepatology.

[5] Paternostro R., Trauner M. (2022). Current treatment of non-alcoholic fatty liver disease. J. Intern. Med..

[6] Loomba R., Friedman S.L., Shulman G.I. (2021). Mechanisms and disease consequences of nonalcoholic fatty liver disease. Cell.

[7] Hu H., Lin A., Kong M., Yao X., Yin M., Xia H., Ma J., Liu H. (2020). Intestinal microbiome and NAFLD: Molecular insights and therapeutic perspectives. J. Gastroenterol..

[8] Albhaisi S.A.M., Bajaj J.S. (2021). The Influence of the Microbiome on NAFLD and NASH. Clin. Liver. Dis..

[9] Han T.R., Yang W.J., Tan Q.H., Bai S., Zhong H., Tai Y., Tong H. (2022). Gut microbiota therapy for nonalcoholic fatty liver disease: Evidence from randomized clinical trials. Front. Microbiol..

[10] Moszak M., Szulinska M., Walczak-Galezewska M., Bogdanski P. (2021). Nutritional Approach Targeting Gut Microbiota in NAFLD-To Date. Int. J. Environ. Res. Public. Health.

[11] Hu J., Ying H., Zheng Y., Ma H., Li L., Zhao Y. (2022). Alanyl-Glutamine Protects against Lipopolysaccharide-Induced Liver Injury in Mice via Alleviating Oxidative Stress, Inhibiting Inflammation, and Regulating Autophagy. Antioxidants.

[12] de Oliveira Santos R., da Silva Cardoso G., da Costa Lima L., de Sousa Cavalcante M.L., Silva M.S., Cavalcante A.K.M., Severo J.S., de Melo Sousa F.B., Pacheco G., Alves E.H.P. (2021). L-Glutamine and Physical Exercise Prevent Intestinal Inflammation and Oxidative Stress Without Improving Gastric Dysmotility in Rats with Ulcerative Colitis. Inflammation.

[13] Liu Z., Huang C., Liu Y., Lin D., Zhao Y. (2018). NMR-based metabolomic analysis of the effects of alanyl-glutamine supplementation on C2C12 myoblasts injured by energy deprivation. RSC Adv..

[14] Araujo R.J., Silva R.G., Vasconcelos M.P., Guimaraes S.B., Vasconcelos P.R., Garcia J.H. (2011). Preconditioning with L-alanyl-glutamine reduces hepatic ischemia-reperfusion injury in rats. Acta Cir. Bras..

[15] Barros M.A., Vasconcelos P.R., Souza C.M., Andrade G.M., Moraes M.O., Costa P.E., Coelho G.R., Garcia J.H. (2015). L-Alanyl-Glutamine Attenuates Oxidative Stress in Liver Transplantation Patients. Transpl. Proc..

[16] Hu J., Zheng Y., Ying H., Ma H., Li L., Zhao Y. (2022). Alanyl-Glutamine Protects Mice against Methionine- and Choline-Deficient-Diet-Induced Steatohepatitis and Fibrosis by Modulating Oxidative Stress and Inflammation. Nutrients.

[17] Polyzos S.A., Kountouras J., Mantzoros C.S. (2019). Obesity and nonalcoholic fatty liver disease: From pathophysiology to therapeutics. Metabolism.

[18] Baeck C., Wehr A., Karlmark K.R., Heymann F., Vucur M., Gassler N., Huss S., Klussmann S., Eulberg D., Luedde T. (2012). Pharmacological inhibition of the chemokine CCL2 (MCP-1) diminishes liver macrophage infiltration and steatohepatitis in chronic hepatic injury. Gut.

[19] Gao W., Xu B., Zhang Y., Liu S., Duan Z., Chen Y., Zhang X. (2022). Baicalin Attenuates Oxidative Stress in a Tissue-Engineered Liver Model of NAFLD by Scavenging Reactive Oxygen Species. Nutrients.

[20] Kobayashi T., Iwaki M., Nakajima A., Nogami A., Yoneda M. (2022). Current Research on the Pathogenesis of NAFLD/NASH and the Gut-Liver Axis: Gut Microbiota, Dysbiosis, and Leaky-Gut Syndrome. Int. J. Mol. Sci..

[21] Recena Aydos L., do Amaral L.A., de Souza R.S., Jacobowski A.C., Dos Santos E.F., Rodrigues Macedo M.L. (2019). Nonalcoholic Fatty Liver Disease Induced by High-Fat Diet in C57bl/6 Models. Nutrients.

[22] Velazquez K.T., Enos R.T., Bader J.E., Sougiannis A.T., Carson M.S., Chatzistamou I., Carson J.A., Nagarkatti P.S., Nagarkatti M., Murphy E.A. (2019). Prolonged high-fat-diet feeding promotes non-alcoholic fatty liver disease and alters gut microbiota in mice. World J. Hepatol..

[23] Gopal S.S., Sukhdeo S.V., Vallikannan B., Ponesakki G. (2023). Lutein ameliorates high-fat diet-induced obesity, fatty liver, and glucose intolerance in C57BL/6J mice. Phytother. Res..

[24] Fang T., Wang H., Pan X., Little P.J., Xu S., Weng J. (2022). Mouse models of nonalcoholic fatty liver disease (NAFLD): Pathomechanisms and pharmacotherapies. Int. J. Biol. Sci..

[25] Song Z., Xiaoli A.M., Yang F. (2018). Regulation and Metabolic Significance of De Novo Lipogenesis in Adipose Tissues. Nutrients.

[26] Li Q., Liao S., Pang D., Li E., Liu T., Liu F., Zou Y. (2022). The transported active mulberry leaf phenolics inhibited adipogenesis through PPAR-gamma and Leptin signaling pathway. J. Food Biochem..

[27] Devereux C.J., Bayliss J., Keenan S.N., Montgomery M.K., Watt M.J. (2023). Investigating dual inhibition of ACC and CD36 for the treatment of nonalcoholic fatty liver disease in mice. Am. J. Physiol. Endocrinol. Metab..

[28] Clifford B.L., Sedgeman L.R., Williams K.J., Morand P., Cheng A., Jarrett K.E., Chan A.P., Brearley-Sholto M.C., Wahlstrom A., Ashby J.W. (2021). FXR activation protects against NAFLD via bile-acid-dependent reductions in lipid absorption. Cell Metab..

[29] Peng A., Liu S., Fang L., Zhu Z., Zhou Y., Yue S., Ma Z., Liu X., Xue S., Qiu Y. (2022). Inonotus obliquus and its bioactive compounds alleviate non-alcoholic fatty liver disease via regulating FXR/SHP/SREBP-1c axis. Eur. J. Pharmacol..

[30] Chen S., Sun S., Feng Y., Li X., Yin G., Liang P., Yu W., Meng D., Zhang X., Liu H. (2023). Diosgenin attenuates nonalcoholic hepatic steatosis through the hepatic FXR-SHP-SREBP1C/PPARalpha/CD36 pathway. Eur. J. Pharmacol..

[31] Di Ciaula A., Passarella S., Shanmugam H., Noviello M., Bonfrate L., Wang D.Q., Portincasa P. (2021). Nonalcoholic Fatty Liver Disease (NAFLD). Mitochondria as Players and Targets of Therapies?. Int. J. Mol. Sci..

[32] Garcia-Ruiz C., Fernandez-Checa J.C. (2018). Mitochondrial Oxidative Stress and Antioxidants Balance in Fatty Liver Disease. Hepatol. Commun..

[33] Zhang C.Y., Liu S., Yang M. (2023). Antioxidant and anti-inflammatory agents in chronic liver diseases: Molecular mechanisms and therapy. World J. Hepatol..

[34] Lee Y.A., Friedman S.L. (2022). Inflammatory and fibrotic mechanisms in NAFLD-Implications for new treatment strategies. J. Intern. Med..

[35] Huang W., Metlakunta A., Dedousis N., Zhang P., Sipula I., Dube J.J., Scott D.K., O’Doherty R.M. (2010). Depletion of liver Kupffer cells prevents the development of diet-induced hepatic steatosis and insulin resistance. Diabetes.

[36] Ryu J., Hadley J.T., Li Z., Dong F., Xu H., Xin X., Zhang Y., Chen C., Li S., Guo X. (2021). Adiponectin Alleviates Diet-Induced Inflammation in the Liver by Suppressing MCP-1 Expression and Macrophage Infiltration. Diabetes.

[37] Aron-Wisnewsky J., Vigliotti C., Witjes J., Le P., Holleboom A.G., Verheij J., Nieuwdorp M., Clement K. (2020). Gut microbiota and human NAFLD: Disentangling microbial signatures from metabolic disorders. Nat. Rev. Gastroenterol. Hepatol..

[38] Liu Y., Xie C., Zhai Z., Deng Z.Y., De Jonge H.R., Wu X., Ruan Z. (2021). Uridine attenuates obesity, ameliorates hepatic lipid accumulation and modifies the gut microbiota composition in mice fed with a high-fat diet. Food Funct..

[39] Tang W., Pan L., Cheng J., Wang X., Zheng L., Wang S., Zhou Y., Wang H. (2022). High-fat-diet-induced gut microbiome changes in mice. Stress. Brain.

