# Supplementary material for: Alanyl-Glutamine Dipeptide Attenuates Non-Alcoholic Fatty Liver Disease Induced by a High-Fat Diet in Mice by Improving Gut Microbiota Dysbiosis
Source: Nutrients. 2023 Sep 14;15(18):3988. doi: 10.3390/nu15183988 (PMC10534574; doi:10.3390/nu15183988)
Supplement: Supplementary file 1 [file nutrients-15-03988-s001.zip › nutrients-2578018-supplementary.pdf]

Supplementary Materials

1. Supplementary Methods

1.1 Analysis of AG in fecal samples

After 12 hours of fasting, Male C57BL/6 mice (aged 6-8 weeks) were orally administered with 1500 mg/kg of AG. The feces were collected at three different time points: prior to administration, 2 hours post-administration, and 6 hours post-administration. AG extraction was performed using a 20% acetonitrile-water solution. After thorough mixing, the supernatant was collected following centrifugation at 2000 g for 3 minutes. All samples were diluted 10 times with a 20% acetonitrile-water solution and filtered through a 0.22 μm aqueous phase syringe filter.

After filtering the samples, analysis was performed using an AB SCIEX Triple Quad™ 4500MD coupled with a Shimadzu Nexera LC-40 liquid chromatograph in Multiple Reaction Monitoring (MRM) mode. The ion pair used was 218.1/130.1. The ion source temperature was set at 500°C, Gas1 was set to 45, Gas2 was set to 65, and the voltage was 5000V. The liquid chromatography was performed using a C18 chromatographic column (Gemini 3 μm, 110 Å, 150×4.6 mm) from Phenomenex. The flow rate was maintained at 0.35 mL/min, and the injection volume was 5 μL. The mobile phase consisted of the following gradient: 0.1% formic acid aqueous solution: acetonitrile = 100:0 (v/v) (0-1 min), 0.1% formic acid aqueous solution: acetonitrile = 80:20 (v/v) (1-5 min), 0.1% formic acid aqueous solution: acetonitrile = 10:90 (v/v) (5-9 min), 0.1% formic acid aqueous solution: acetonitrile = 10:90 (v/v) (9-12 min), 0.1% formic acid aqueous solution: acetonitrile = 100:0 (v/v) (12-12.5 min), 0.1% formic acid aqueous solution: acetonitrile = 100:0 (v/v) (12.5 min-15 min). The concentration of the AG standard solution was 100 ng/mL.

2. Supplementary Tables and Figures

2.1 Supplementary Table S1. Composition of the standard diet (SD) and HFD diet formulas

Formulas	SD		HFD	
	gm%	kcal%	gm%	kcal%
Protein	19.2	20	26	20
Carbohydrate	67.3	70	26	20
Fat	4.3	10	35	60
Kcal/gm	3.85		5.24	
Total		100		100
**Ingredient**	**gm**	**kcal**	**gm**	**kcal**
Casein, 80 Mesh	200	800	200	800
L-Cystine	3	12	3	12
Corn Starch	506.2	2024.8	0	0
Maltodextrin 10	125	500	125	500
Sucrose	68.8	275.2	68.8	275.2
Cellulose	50	0	50	0
Soybean Oil	25	225	25	225
Lard	20	180	245	2205
Mineral Mix S10026	10	0	10	0
Dicalcium Phosphate	13	0	13	0
Calcium Carbonate	5.5	0	5.5	0
Potassium Citrate, 1 H_2O	16.5	0	16.5	0
Vitamin Mix V10001	10	40	10	40
Choline Bitartrate	2	0	2	0
Total	1055.05	4057	773.85	4057

2.2 Supplementary Figure S1

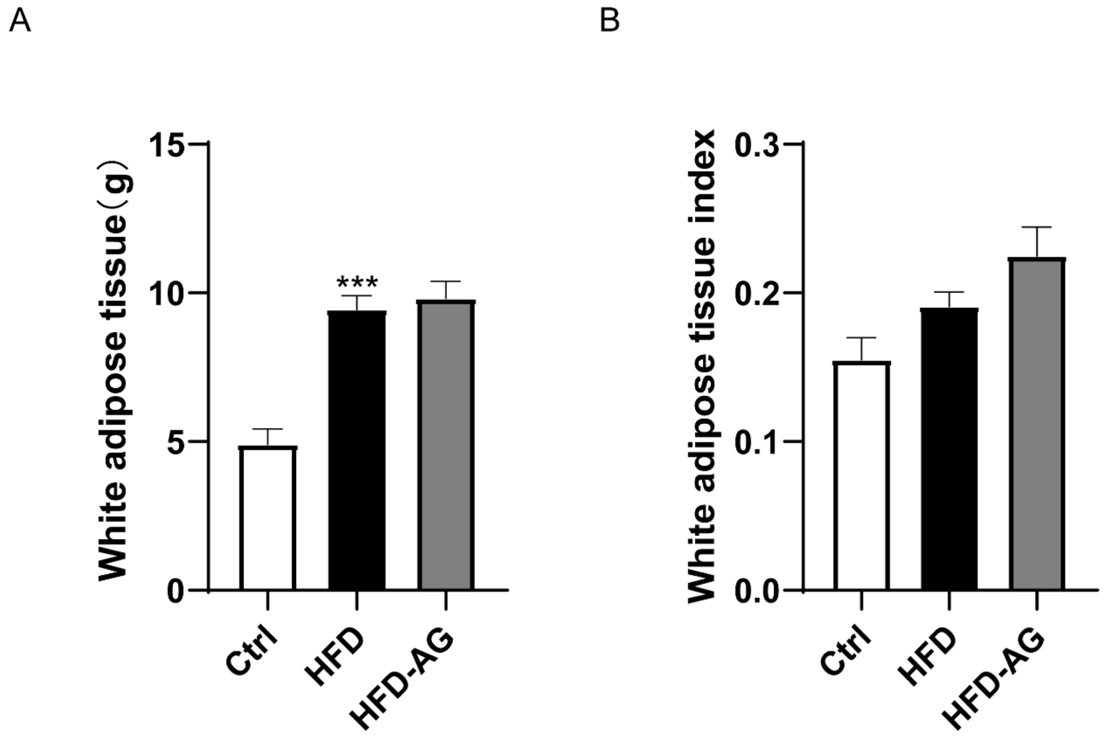

Figure S1. Effects of AG on the white adipose tissue weight and white adipose tissue index in HFD feeding mice. (A) White adipose tissue weight; (B) White adipose tissue index. Results are expressed as the mean ± SEM. *** $P < 0.001$, compared to the Ctrl group.

2.3 Supplementary Figure S2

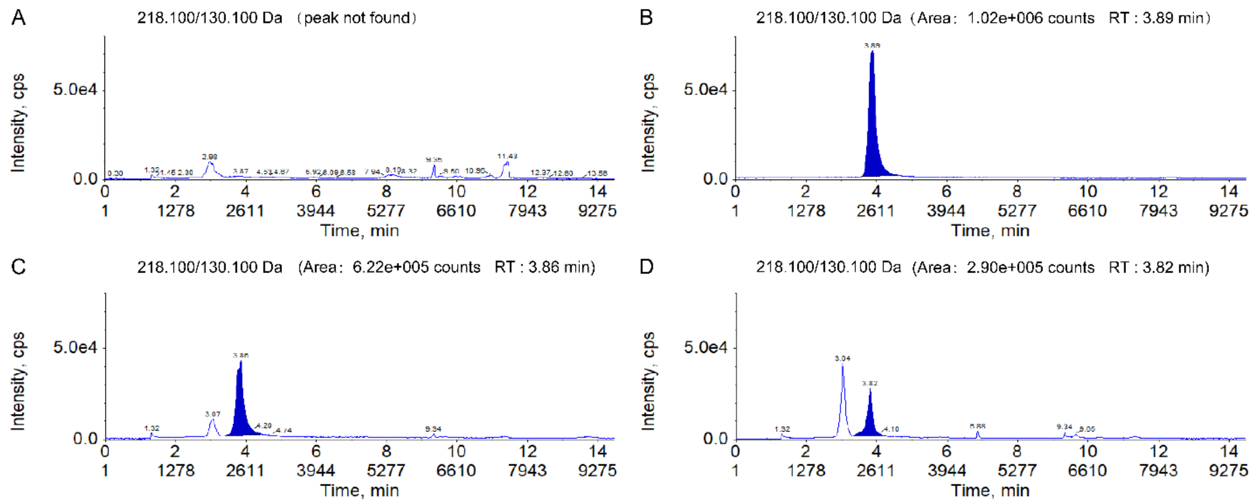

Figure S2. LC-MS analysis of AG in fecal samples. (A) Fecal samples collected from mice before administration; (B) 100 ng/mL AG standard solution. (C) Fecal samples collected from mice 2 hours after AG administration; (D) Fecal samples collected from mice 6 hours after AG administration. The retention time for the 100 ng/mL AG standard was 3.89 minutes, with a peak area of $1.02e^{6}$. AG was not detected in the fecal samples collected before administration. In the fecal samples collected 2 hours after AG administration, AG was detected with a peak area of $6.22e^{5}$, thus the AG content in mouse feces is calculated to be 6.098 μg/g of feces. In the fecal samples collected 6 hours after AG administration, AG was detected with a peak area of $2.9e^{5}$, indicating an AG content of 2.843 μg/g of feces.
